# Vibrotactile speech cues are associated with enhanced auditory processing in middle and superior temporal gyri

**DOI:** 10.1038/s41598-025-07718-8

**Published:** 2025-07-12

**Authors:** Alina Schulte, Jeremy Marozeau, Andrej Kral, Hamish Innes-Brown

**Affiliations:** 1 Department of Experimental Otology of the Clinics of Otolaryngology, Institute for AudioNeuroTechnology (VIANNA), Hannover Medical School, Hannover, Germany; 2 Eriksholm Research Center, Oticon A/S, Snekkersten, Denmark; 3 Music and Cochlear Implants Lab, Department of Health Technology, Technical University of Denmark, Kongens Lyngby, Denmark; 4 Department of Basic Neurosciences, University of Geneva, Geneva, Switzerland; 5 Australian Hearing Hub, School of Medicine and Health Sciences, Macquarie University, Sydney, Australia; 6 Hearing Systems Section, Department of Health Technology, Technical University of Denmark, Kongens Lyngby, Denmark

**Keywords:** Functional near-infrared spectroscopy, Multisensory processing, Multimodal speech, Auditory processing, Audio-tactile perception, Auditory system, Cognitive neuroscience, Sensory processing, Somatosensory system

## Abstract

Combined auditory and tactile stimuli have been found to enhance speech-in-noise perception both in individuals with normal hearing and in those with hearing loss. While behavioral benefits of audio-tactile enhancements in speech understanding have been repeatedly demonstrated, the impact of vibrotactile cues on cortical auditory speech processing remains unknown. Using functional near-infrared spectroscopy (fNIRS) with a dense montage setup, we first identified a region-of-interest highly sensitive to auditory-only speech-in-quiet. In the same region, we then assessed the change in activity ('audio-tactile gains’) when presenting speech-in-noise together with a single-channel vibratory signal to the fingertip, congruent with the speech envelope’s rate of change. In data from 21 participants with normal hearing, audio-tactile speech elicited on average 20% greater hemodynamic oxygenation changes than auditory-only speech-in-noise within bilateral middle and superior temporal gyri. However, audio-tactile gains did not exceed the sum of the unisensory responses, providing no conclusive evidence of true multisensory integration. Our results support a metamodal theory for the processing of temporal speech features in the middle and superior temporal gyri, providing the first evidence of audio-tactile speech processing in auditory areas using fNIRS. Top-down modulations from somatosensory areas or attention networks likely contributed to the observed audio-tactile gains through temporal entrainment with the speech envelope’s rate of change. Further research is needed to understand the neural responses in concordance with their behavioral relevance for speech perception, offering future directions for developing tactile aids for individuals with hearing impairments.

**Supplementary Information:**

The online version contains supplementary material available at 10.1038/s41598-025-07718-8.

## Introduction

Sensory information from non-auditory modalities, such as visual cues from a speaker’s face, plays a significant role in auditory speech processing^1^. This becomes particularly important in noisy listening environments and for individuals with hearing loss, where auditory cues are less reliable. Multimodal speech information enhances speech perception and improves intelligibility^2,3^ by providing additional a priori information in a Bayesian sense^4^. The broadband envelope has been identified as a crucial speech feature for successful speech understanding^5–8^ and is also reflected in lip and mouth movements^9,10^. Consequently, slow temporal fluctuations of speech appear to enhance intelligibility even when presented non-auditorily^11^. When delivered as vibrotactile cues to the skin, such as on the fingertip or wrist, congruent envelope cues have been found to improve understanding of speech in noise in people with normal hearing thresholds as well as cochlear implant users^6,12–20^. These findings suggest novel rehabilitation opportunities for individuals with hearing loss, underscoring the need for a deeper understanding of the neural encoding and integration of tactile stimuli into speech processing.

Audio-visual^21–23^ and visual-only speech processing^24–26^ have both been localized to auditory areas of the brain. These findings support an amodal perspective of speech processing in temporal areas^27^, showing that separate unisensory processing is not necessarily required prior to convergence. Integration of multimodal speech cues likely occurs at multiple processing stages, including areas beyond auditory cortices^28,29^—a hypothesis further supported by recent evidence from a large-scale meta-analysis^30^. Several brain regions have been linked to speech perception, and theories on the contribution of, for example, the motor system, have been extensively discussed^31,32^. Recent frameworks also suggest a role for the somatosensory system in the neural processing of speech^33^, offering a promising perspective for the use of vibrotactile speech information.

Different psychophysiological methods have been used to examine how vibrotactile speech support affects auditory speech processing. Electroencephalography (EEG) research has shown that vibrotactile cues aligned with the syllabic rate of speech entrain theta-range oscillatory neural activity^34^, and that phase alignment of amplitude modulations between auditory noise and electrotactile carrier signals is critical for enhancing auditory steady-state responses^35^. Multisensory gain has also been observed in temporal response functions (using forward modelling of EEG) for synchronously combined audio-tactile speech stimuli, likely originating in auditory cortices^18^. A follow-up study using similar vibrotactile pulses at 5 Hz found that responses were sustained even beyond the stimulation period^36^. Together, these findings suggest that tactile input supports the segmentation of auditory speech into syllables, possibly mediated by a phase-resetting mechanism^37^. Moreover, a functional magnetic resonance imaging (fMRI) study demonstrated activation of superior temporal gyri (STG) in response to continuous tactile speech stimuli, but not to token-based stimuli^38^. These results support the hypothesis that vibrotactile enhancement engages auditory cortical regions and highlight the importance of preserving the temporal structure of speech in the tactile signal to elicit auditory-like processing. While EEG offers high temporal resolution for assessing the impact of tactile stimuli on oscillatory dynamics, its spatial resolution is limited and relies on source localization algorithms to estimate the origin of neural signals. In contrast, fMRI provides high spatial resolution but requires a costly, stationary setup with a loud acoustic environment that poses challenges for auditory experiments. Both methods are prone to artifacts from hearing devices, rendering them suboptimal for studying individuals with hearing loss—those who may benefit most from vibrotactile speech support. To overcome these limitations, the present study employs functional near-infrared spectroscopy (fNIRS) to investigate whether previously reported tactile enhancements of auditory speech processing are reflected in amplitude differences in evoked cortical hemodynamic responses. While fNIRS represents a compromise in spatial and temporal resolution, it provides sufficient sensitivity to detect speech-evoked cortical responses from optodes positioned over temporal regions, rather than reflecting summed or distant sources as in EEG. Additionally, it is quiet, easy to set up, and compatible with hearing aids and cochlear implants, without causing interfering artifacts^39^.

### fNIRS as a method for studying cortical speech-related hemodynamics

fNIRS is increasingly popular in hearing and language research^40–43^. It measures relative concentration changes in oxygenated (HbO) and deoxygenated (HbR) hemoglobin in superficial cortical tissue, which indirectly reflect neural activity through neurovascular coupling. fNIRS enables the identification of cortical sources of neural activation, making it well-suited to pinpoint superficial cortical sites involved in auditory speech processing. Numerous fNIRS studies have reported bilateral temporal and left inferior frontal cortical activations associated with different aspects of speech processing^44–51^, and have identified differences in activation patterns between participants with natural hearing and those with cochlear implants^52–54^. Cross-modal activations have been observed across different participant groups^55–57^ and this cross-modal activation has been linked to speech perception outcomes in cochlear implant users; however, the direction of these associations has varied^58,59^. Evidence of multisensory integration of speech stimuli using fNIRS remains elusive^60,61^.

Not all studies have consistently observed robust auditory activations. Factors such as participants’ vigilance, vascular confounds (e.g. blood-stealing^62^) or the choice of baseline^48^ have been proposed as possible explanations. Auditory fNIRS responses predominantly reflect activity from the auditory belt and association cortices, as the primary auditory cortex lies deep within the lateral sulcus and is reached with less than 1% specificity^63^. The isolation of neural auditory activity is further complicated by significant contributions from physiological sources, including activity from the muscle temporalis and hemodynamic changes in extracranial vessels in temporal regions.

As a result, auditory fNIRS experiments require careful consideration of experimental design and analysis methods^64^. For instance, signal enhancement algorithms have been found essential for data cleaning and extracting auditory responses^65^. Additionally, approaches to maximize individual responses by determining each participant’s best-responding channels prior to group-level analyses have been applied^57,58^.

### Aims of the current study

The central aim of the present study was to examine whether vibrotactile speech cues influence auditory cortical responses to speech, as measured with fNIRS. To address this question, we adopted a two-step approach.

We first identified fNIRS channels that consistently responded to auditory speech-in-quiet stimuli. This step enabled the definition of a data-driven region-of-interest (ROI), which served as the basis for the subsequent analysis in the present study and will be applicable in future studies using a similar montage setup. Second, we compared hemodynamic responses to audio-tactile speech-in-noise with those to auditory speech-in-noise within the previously identified auditory ROI. This comparison aimed to determine whether vibrotactile cues enhance auditory processing. Additionally, we evaluated whether the observed audio-tactile responses exceeded the linear sum of the unisensory responses, a conventional criterion for identifying multisensory integration^66^.

## Material and methods

### Participants

Twenty-one normal-hearing adults (8 females, 13 males; mean age = 35 years, SD = 9) were included in the analysis. Data from four additional participants were excluded due to insufficient data quality, as more than 50% of their fNIRS channels had to be rejected based on the criteria described below (see in “Preprocessing” section). All participants were recruited from among colleagues at Oticon or the Technical University of Denmark. They were right-handed, had normal hearing, and were highly proficient in English, but not native speakers. Previous research has reported the largest multisensory benefits in participant groups who are non-native speakers of the language used for stimulus presentation^16,17,67–69^. Ethical approval was obtained from the Danish Science-Ethics Committee (reference H-16036391). The experiment was conducted in accordance with the respective guidelines and regulations, and all participants provided written informed consent.

### Equipment

**Fig. 1:**
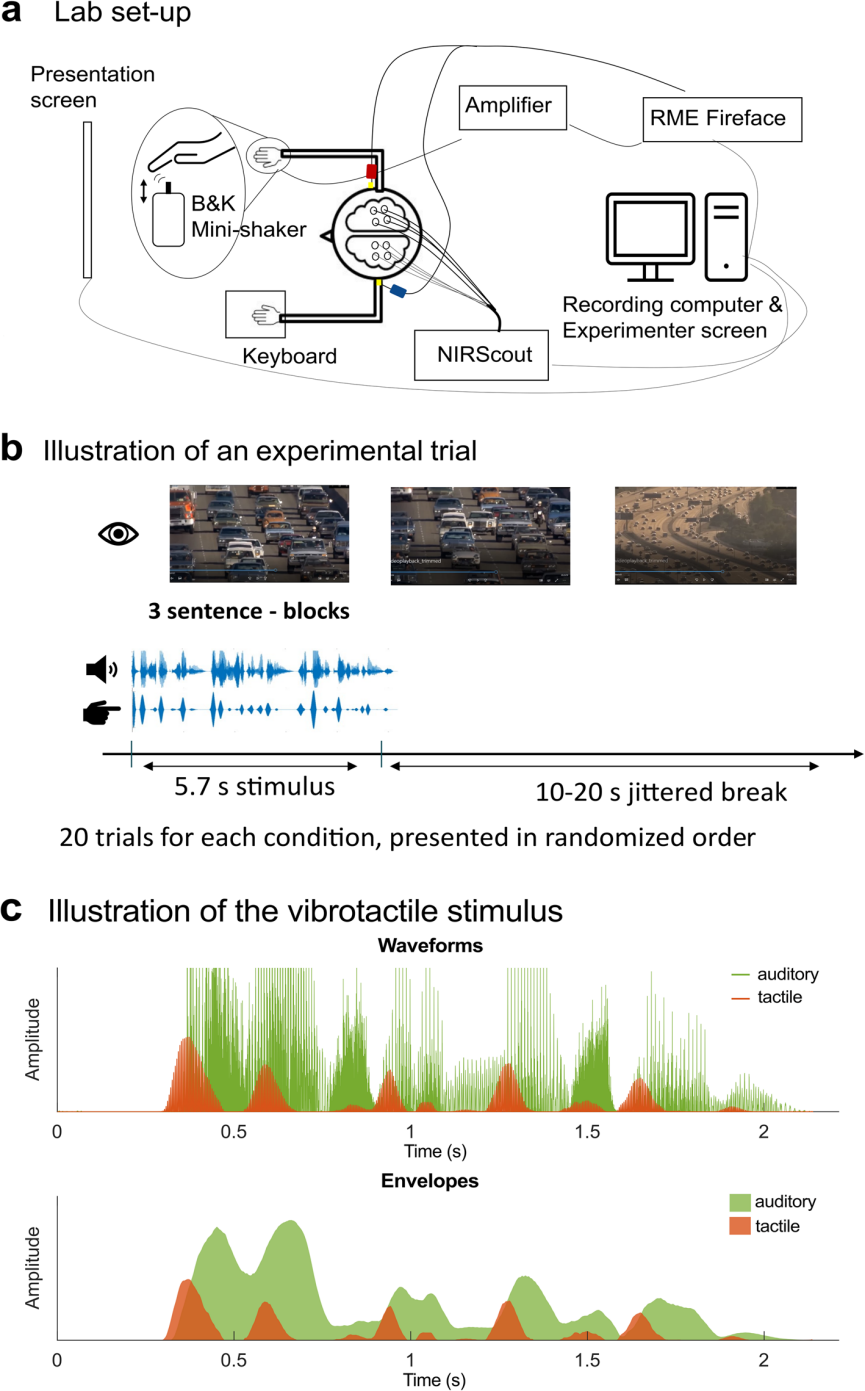
Experimental setup and paradigm. (**a**) Schematic illustration of a participant in the lab receiving auditory stimuli through insert earphones and vibrotactile stimulation on the right index fingertip. (**b**) One trial of the experimental procedure including auditory, tactile, and visual stimulus presentations. (**c**) Auditory and vibrotactile signals corresponding to the sentence “The house had a nice garden”. The upper panel shows rectified waveforms of the auditory and tactile stimuli. The tactile stimulus (in orange) is a 230 Hz carrier modulated by the rate of change of the auditory envelope. The bottom panel displays area-filled envelopes of the auditory and tactile speech signals. Note that in the experiment, three sentences were always presented consecutively, forming one stimulus block, as illustrated in (**b**).

The study was conducted in a sound-treated booth at Eriksholm Research Center, Denmark. Auditory stimuli were presented diotically via Etymotic ER-2 insert earphones (Etymotic Research, Illinois, USA). Tactile stimuli were delivered through a Bruel and Kjær minishaker type 4810 equipped with an accelerometer (B&K 4533) featuring a circular contact area of 12 mm in diameter, positioned under the participant’s right index fingertip. Stimulus files were converted by an RME Fireface UCX audio interface, with one output channel for each signal (auditory, tactile). These outputs were connected to separate amplifiers, which drove the earphones and minishaker, respectively (Fig. 1a).

Acceleration of the tactile probe was measured from the accelerometer, which also served as the contact surface for stimulus presentation. The accelerometer was connected to a B&K 1704 CCLD conditioner, which in turn was connected to the input of the RME Fireface soundcard. Sound intensities were measured using a B&K sound level meter type 2250, along with an ear simulator type 4157, and the external ear accessory DB 2012.

Cortical oxygenation changes were recorded using an fNIRS system (NIRx NirScout) with 16 LED sources and 16 avalanche photodiode detectors. Measurements targeted temporal, inferior-frontal and parietal regions (see Fig. 3 for the probe layout). Near-infrared light at 760 nm and 850 nm wavelengths was sampled at a rate of 3.9063 Hz using the NIRStar 15.3 acquisition software (NIRx Medizintechnik GmbH, Berlin, Germany).

### Stimuli

The stimuli consisted of English HINT sentences presented in nine different conditions, of which only five were relevant for the present investigation: *auditory speech-in-quiet*, *auditory speech-in-noise*, *audio-tactile speech-in-noise*, *tactile speech,* and a silent *control* condition. Each condition comprised 20 trial repetitions with silent inter-stimulus intervals. A sixth condition (*auditory noise-in-quiet*) was disregarded in the present analysis. In addition to these six conditions with silent inter-stimulus intervals, three further conditions involving continuous background noise were recorded but excluded from analysis as they were not relevant for the research questions of this study. These were: S*peech-in-continuous background noise*, *tactile speech-in-continuous background noise* and a second control condition with *continuous background* noise only.

Stimuli were grouped in eight experimental blocks, with optional breaks between blocks. Blocks 3 and 6 included the conditions with continuous background noise, presented in random order. The remaining blocks comprised the six conditions with silent background between stimuli, also played in random order. During all experimental blocks, a silent movie [Koyaanisqatsi (Godfrey Reggio^70^)], unrelated to the speech stimuli, was shown continuously on the computer screen to keep participants engaged. Including breaks, the listening task lasted approximately 70 min.

#### Auditory stimuli

Speech stimuli consisted of three concatenated sentences, resulting in trial durations between 5.56 and 5.7 s. In the *auditory speech-in-noise* and *audio-tactile speech-in-noise* conditions, noise started playing 0.5 s before speech onset and continued until the end of the speech signal. For the remaining conditions, 0.25 s pauses were inserted between sentences to achieve comparable stimulus lengths.

For the two speech-in-noise conditions, speech-shaped noise with an identical long-term average spectrum as the speech material was generated and presented at a fixed signal-to-noise ratio of − 2 dB for all participants. Given that participants were not native English speakers, and based on previous investigations showing little variance in speech reception thresholds for normal-hearing participants, it can be assumed that the presented speech stimuli were intelligible at approximately 50–70%. All auditory stimuli were equalized to have the same root-mean-square and presented at a level of 73.8 dB SPL LAF.

#### Tactile stimulus

The *tactile speech* stimulus was based on the sentence envelope’s rate of change, which modulated a single carrier signal. As a consequence, the peaks of the tactile signal align with syllable onsets rather than the centers of syllables, as would be the case when using the original broadband envelope (Fig. 1c). This tactile stimulus differs from our previous study^19^, in which we used a 4-band tactile stimulus that was summed into a single output, based on Fletcher et al.^13^. The change to a simpler, single-carrier tactile signal cueing the envelope’s peak rate was inspired by an EEG study revealing that neurons in the superior temporal gyrus (STG) encode amplitude changes rather than absolute amplitude of speech broadband envelopes^71^. In a pilot experiment with seven participants, no differences in effectiveness between the old and the new tactile speech stimuli on speech enhancement were identified. Nonetheless, the finding that very sparse tactile signals aligned with phonemes elicited similar enhancement effects in another study^18^ further supported our decision to switch to a simpler stimulus.

To generate the tactile envelope signal we followed the procedure from MacIntyre et al.^72^, using their env3-function to extract the envelope^73^, followed by taking its first derivative, smoothing, rectifying and rescaling. The resulting envelope rate of change was then used to modulate a carrier signal of 230 Hz—a frequency to which the Pacinian corpuscles in glabrous skin are highly sensitive^74^. Tactile stimuli were presented with an average displacement of 3.7 μm to the right fingertip (dominant hand for all participants). We used a stronger presentation intensity compared to our previous investigation^19^, in which detection threshold estimates were around 0.1 μm for a 230 Hz carrier signal and stimulus presentation was at 1.4 μm displacement on average. Hence, stimulus intensity was most likely well above threshold in the current study as was confirmed subjectively by all participants during the experiment.

All stimuli were generated prior to data collection using MATLAB (Version R2020a, Natick, Massachusetts, The MathWorks Inc.) and presented during the experiment using PsychoPy3^75^. Event triggers were sent from PsychoPy to NirStar using LabStreamingLayer^76^ at the start of stimulus presentation. The tactile and auditory signals were presented simultaneously, as they were stored in the same two-channel file that was sent to the respective playback devices (earphones and minishaker).

### Experimental design and procedure

At the beginning, participants were informed about the experimental procedure and provided written informed consent. If no audiogram had been conducted within the last 12 months, pure-tone audiometry at 0.25, 0.5, 1, 2, 4 and 8 kHz was performed to ensure participants could be classified as normal-hearing based, on a mean threshold below 20 dB HL. Subsequently, participants were equipped with the fNIRS cap and instructed to avoid head movements and contractions of facial muscles. A passive speech perception paradigm followed, during which participants were instructed to attend to the stimuli, although no active task had to be performed. Between experimental blocks, participants were asked how well they could understand the speech-in-noise stimuli to remind them to direct attention to the stimuli. Trials of the experimental conditions occurred in random order, interleaved with a jittered break of 10–20 s. The trial order and stimuli were identical across all participants (see Fig. 1b for an example trial).

### fNIRS montage

A suitable optode placement targeting speech-relevant areas was created using NIRSite 2021.4 (NIRx Medizintechnik GmbH, Berlin, Germany). Source-detector pairs were formed based on specificity information from the fNIRS Optodes’ Location Decider (fOLD) toolbox^63^, using the Juelich brain parcellation atlas. Optodes covered auditory and speech processing areas in the temporal lobes, as well as the left inferior frontal gyrus and somatosensory cortex. Most fNIRS studies consider EEG 10–10 locations as possible positions for fNIRS optodes. As one of the study’s aims was to localize auditory responses as precisely as possible, spatial resolution was increased by adding channels between the 10–10 locations. Note that the additional optode positions are not equivalent to the 10–5 system, in which positions are defined at 5% steps from nasion to inion. Instead, the additional optodes remained spaced at 10% intervals from nasion to inion, while being placed at 5% distances between existing optodes along the preauricular-to-preauricular axis. The denser grid enabled the addition of diagonal channel connections to the montage, which would otherwise exceed a suitable optode distance. The final montage included a total of 49 long channels with source-detector distances ranging from 2.1 to 4.3 cm. In addition, eight short-channel detectors were included in the montage at a distance of 0.8 cm from their respective sources to capture scalp hemodynamics for signal correction (see Fig. 3).

### Data analysis

Data were analyzed using the Python-based toolbox MNE-NIRS, including MNE^77^, MNE-NIRS (version 1.5.1^47^) Nilearn^78^ and statsmodels^79^. Cortical activation was inferred from the response magnitudes of HbO signals, estimated using a waveform averaging analysis and a general linear model (GLM). While GLMs address the statistical properties of fNIRS data more appropriately and are generally expected to yield more accurate results, waveform visualizations offer a more intuitive and conventional representation of the data, facilitating interpretation and understanding. Rather than comparing the two analysis methods directly, we interpret converging results from both as an indicator of robust auditory responses, with cross-validation between approaches increasing the credibility of our findings.

#### Preprocessing

For each of the two wavelengths, the recorded traces of raw light intensities were converted to optical density (i.e., transformation to a logarithmic scale with zero mean). To identify channels with insufficient data quality, that are unlikely to reflect cerebral oxygenation changes, we considered scalp coupling indices (SCI) and peak power (cross-correlation of wavelengths 1 and 2 and its power spectral density within the cardiac range as, described by Pollonini et al.^80^), as well as each channel’s standard deviation (SD). Gaps larger than 30 s between segments from − 5 to 15 s around stimulus onset were discarded for this step, ensuring data quality assessment was based on experimental blocks only (i.e., not including break periods of the experiment). Channels were excluded from analysis if they met one or more of the following criteria: SCI < 0.7, more than 50% of 10 s segments with a peak power < 0.1, or a SD > 0.2 (equivalent to a coefficient of variation of 20%). These criteria led to the exclusion of, on average, 2.2 out of 49 long channels (SD = 3.6 channels) and 1.4 out of 8 short channels (SD = 2.2 channels) per participant (see Fig. S1). After channel rejection, temporal derivative distribution repair^81^ was applied to diminish motion artifacts. Corrected optical density data were converted to hemoglobin concentration changes by applying the modified Beer–Lambert law with a partial pathlength factor of 0.1. The hemoglobin data were low-pass filtered using a 5th order IIR Butterworth filter with a cut-off frequency of 0.25 Hz, and then high-pass filtered by application of the same filter with a high-pass cut-off frequency of 0.005 Hz.

Finally, signal quality was further enhanced using the correlation-based signal improvement method^82^. This technique must be treated with caution, as it transforms the data substantially and relies on the assumptions that HbO and HbR signals are positively correlated during motion artifacts and (perfectly) negatively correlated otherwise—premises that do not fully hold. For instance, a slightly delayed dip in the HbR signal relative to the HbO peak is expected and these relationships are likely to vary across individuals and brain regions. Correlation-based signal improvement was included in our preprocessing pipeline only after evaluating its effect on the contrast-to-noise ratio (CNR, defined as in Zhou et al.^83^) of waveform averages, comparing all stimulus conditions combined versus no stimulus (control conditions). Note that this response was not of interest for our main analysis. Comparing the CNR before and after applying correlation-based signal improvement, we found that the method significantly enhanced detection of the hemodynamic response to stimuli without altering the response to the control stimuli (see Fig. S2). The algorithm computes a new HbO signal based on both original chromophore signals, whereas the new HbR signal is entirely derived from the corrected HbO. Consequently, the corrected HbR signal does not contain any independent information after this processing step. For visualization purposes, both chromophores are displayed in waveform averages throughout the manuscript, however, statistical analyses and interpretations are based solely on the corrected HbO signal.

To remove contributions from scalp hemodynamics, we performed a separate GLM using all available short-channel traces as regressors (up to 8 HbO and 8 HbR traces). In one participant, this step was omitted because no short channels with sufficient data quality were available. Previous studies have demonstrated the effectiveness of including either all available short channels or their principal components in the model^84,85^ and recommend performing short-channel correction in a separate step to reduce collinearity between regressors^85,86^. We found that this approach considerably increased the CNR compared to the alternative method implemented in MNE-NIRS (i.e., subtracting a scaled version of the nearest short channel based on Fabbri et al.^87^, Saager and Berger^87^ and Scholkmann et al.^88^, see Fig. S2).

#### Single participant feature extraction: GLM and waveform averaging

##### GLM analysis

Beta-values, reflecting the amplitude of the modelled hemodynamic response, were estimated for each participant. Regressors in the design matrix were generated by convolving a Glover hemodynamic response function (HRF)^89^ with a boxcar function of 3.4 s duration. This deviation from the actual stimulus duration was applied following Luke et al.^47^, who found that a boxcar duration 1.67 times shorter than the actual stimulus length best captured the hemodynamic responses in a similar in a passive auditory listening paradigm. Additional cosine drift regressors were included to account for drifts not removed by the prior high-pass filtering. The high-pass cut-off frequency for the drift model was defined based on the longest inter-stimulus interval between two conditions of interest. An autoregressive model of order 5 was used to estimate beta-values for each channel, weighting each regressor to explain the maximum variance in the data.

##### Waveform averaging analysis

As a second measure, stimulus-evoked neural responses were estimated for each participant using a waveform averaging analysis. Channels were segmented into epochs from − 5 to 20 s relative to stimulus onset and baseline-corrected by subtracting the mean of the pre-stimulus period. Additionally, a linear detrend was applied to each epoch. Epochs with HbO peak-to-peak amplitudes exceeding 45 Δμmol/l were rejected. On average, this threshold led to the exclusion of 0.6% of epochs per participant (SD = 14.5%, median = 4.3%).

To reduce the waveform to a single value suitable for statistical analysis, the mean HbO signal between 5 and 8 s post-stimulus onset was calculated (referred to as the waveform mean amplitude). This time window corresponds to the expected peak of the hemodynamic response (around 6.5 s after stimulus onset, considering a Glover HRF convolved with a boxcar function of 3.4 s length).

#### Generation of data-driven auditory region-of-interest

To generate a ROI containing channels sensitive to auditory speech processing, single-participant results for each channel in the *auditory speech-in-quiet* condition were analyzed. For each participant, the ten channels showing the highest HbO responses were identified. Then, for each channel, the number of participants for which it appeared among the top ten was counted. This process was performed separately for both waveform mean amplitudes and beta-values. The final data-driven ROI was defined as the intersection of the top-ranked channels from the two top ten lists. This procedure revealed six left and right temporal channels which were defined as the *auditory-ROI* (see Fig. 3). To compute the ROI response, amplitude estimates (waveform mean amplitudes and beta-values, respectively) were averaged with equal weighting.

#### Group level analysis

Single participant results were summarized using linear mixed-effect models (LMMs) to determine group HbO responses for individual condition activations, as well as to test our hypothesis of potentially stronger responses for *audio-tactile speech-in-noise* compared to *auditory speech-in-noise* within the identified *auditory-ROI*.

Separate models were conducted for beta-values and waveform mean amplitudes. Within each model, multiple comparisons were accounted for using the Benjamini–Hochberg procedure to control the false discovery rate (FDR) as implemented in the statsmodels package^79^. This method uses an accepted false discovery rate of 0.05 to determine a *p* value threshold for rejecting hypotheses, based on the rank and total number of comparisons (i.e., the number of conditions multiplied by the number of channels or ROIs). We considered the agreement of results from both GLM and waveform-averaging analyses as an indicator of the robustness of the overall HbO results and their relevance for interpretation.

##### Condition activations

To initially display the data, HbO responses for each condition were compared to baseline with an interaction of channels and condition as main effect, participants as a random effect and a suppressed intercept (in Roger–Wilkinson notation: HbO response ~ − 1 + Condition:channel + (1|Participant)). To obtain ROI results instead of single-channel activations, the model was repeated with Condition as the main effect, using each participant’s auditory-ROI activation as input instead of single channel results.

##### Contrasts

To test for the presence of audio-tactile gains in the *auditory-ROI*, an LMM with *Condition* as the main effect and *Participants* as a random effect was conducted, comparing *audio-tactile speech-in-noise* against *auditory speech-in-noise* in the *auditory-ROI* (Δ HbO response ~ Condition + 1|Participant). By not suppressing the intercept, the first level of the Condition factor (auditory speech-in-noise) serves as a reference category, and the resulting coefficient for the audio-tactile condition represents the contrast between the two conditions. If significant audio-tactile gains were present, their effect size was compared to each participant’s sum of the unimodal activations to assess multisensory integration: an additional LMM, including the conditions *audio-tactile speech-in-noise* and an auxiliary condition comprising the sum of the two unisensory activations (*auditory speech-in-noise* and *tactile speech*), was conducted.

## Results

### Condition activations

#### Single channel results

**Fig. 2:**
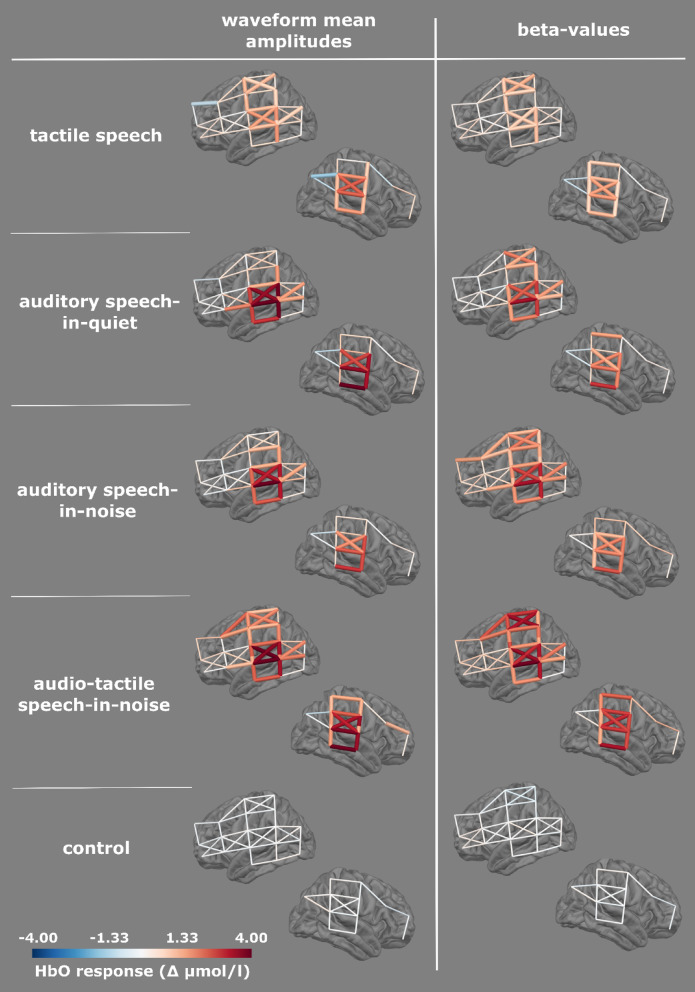
Single channel group results. HbO waveform mean amplitudes and beta-values for all experimental conditions are displayed in lateral views of the left and right hemisphere. Responses are displayed as colored tubes, in the respective position of the montage. Thicker tubes represent significant channels after FDR correction.

Group results for single-channel activations across task conditions revealed significant positive HbO responses predominantly in temporal areas for all conditions except for the *control* (Fig. 2). Activation patterns were overall similar for beta-values and waveform mean amplitudes, with significant activations observed in auditory regions. However, they differed in somatosensory channels: beta-values revealed significant activations in channels over the left primary somatosensory cortex across all stimulus conditions (most pronounced for *audio-tactile speech-in-noise*), while waveform mean amplitudes indicated left primary somatosensory cortex activation selectively for *tactile speech* and *audio-tactile speech-in-noise*.

#### ROI results

**Fig. 3:**
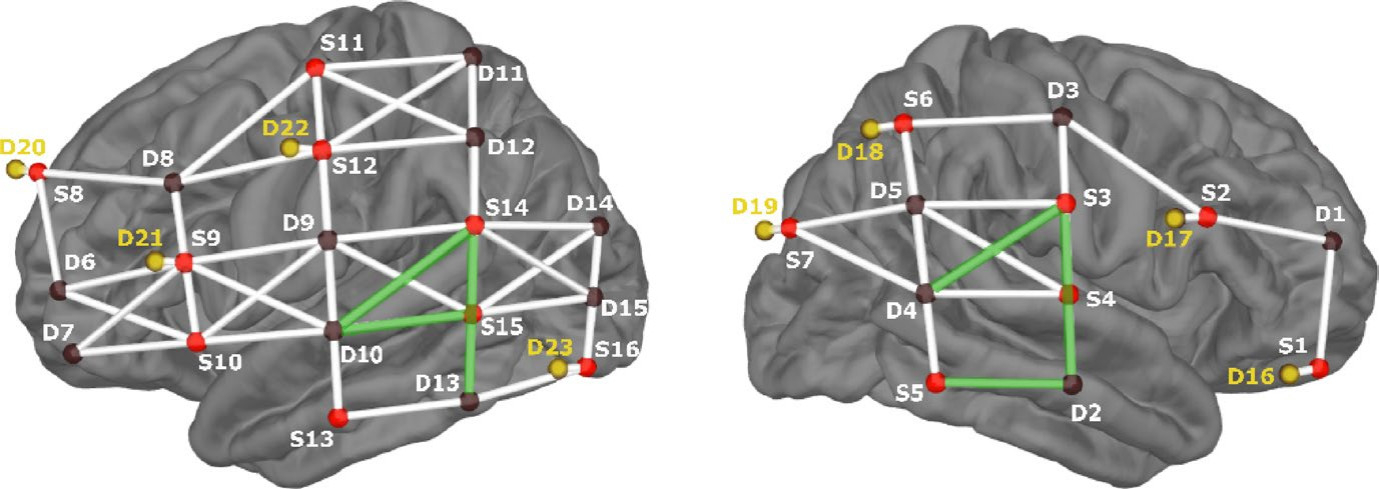
Montage with auditory-ROI. Lateral views of left and right hemispheres. Red circles indicate the positions of sources, and brown circles the positions of detectors. Short-channel detectors are highlighted in yellow. Channels between optodes are displayed in white, except for those forming the auditory ROI, which are displayed in green.

Based on consistent *auditory speech-in-quiet* responses across participants, an *auditory-ROI* was defined, comprising six channels positioned over the left and right middle and superior temporal gyri (Fig. 3, Table 1).

**Table 1:** Auditory-ROI: specificity of underlying targeted brain regions.

Region	Channels	EEG locations	Specificity
Data-driven *auditory-*ROI	S3-D2	C6-T8	54% rSTG, 31% rMTG
	S5-D2	TP8-T8	63% rMTG, 35% rIFG
	S3-D4	C6-TP8h	rMTG, rSTG
	S14-D13	CP5-TP7	72% lMTG, 17% lSTG
	S14-D10	CP5-T7h	*lMTG, lSTG*
	S15-D10	TP7h-T7h	*lMTG, lSTG*

Specificity information on cortical structures targeted byc each channel of the auditory-ROI is provided when available, based on the fOLD toolbox^63^. The montage included additional channels beyond the 10–10 system for which no specificity data are available.

*STG* superior temporal gyrus, *MTG* middle temporal gyrus, *r* right, *l* left.

**Fig. 4:**
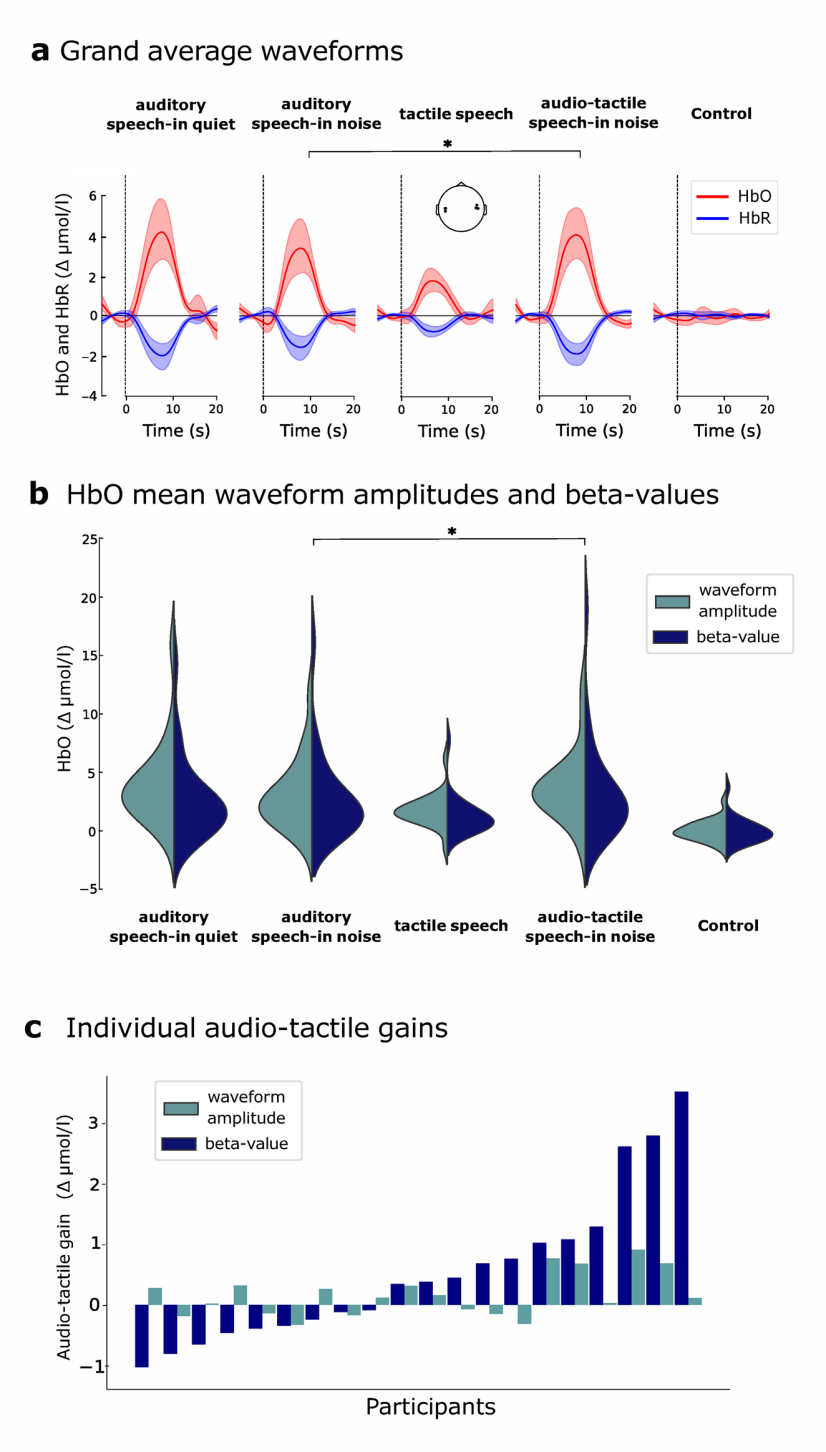
ROI results. (**a**) Grand average waveforms for each task condition in the auditory-ROI. HbO traces are displayed in red, HbR traces in blue, with shaded areas representing 95% confidence intervals. Both chromophores are displayed for illustrative purposes, but only HbO was analyzed statistically. (**b**) Distributions of HbO waveform mean amplitudes and HbO beta-values in the auditory-ROI are shown for each task condition using split violin plots. (**c**) Individual audio-tactile gains in the auditory-ROI. Each participant’s audio-tactile gain, as derived from the GLM and the waveform averaging analysis is shown in a bar plot, sorted by size based on the GLM-derive.

In line with the activation patterns observed in single channel results (Fig. 2), the ROI group results also showed significant responses for all task conditions except the *control* (Fig. 4a,b; Table 2). *Tactile speech* reached significance only in the waveform averaging analysis (Table 2). Overall, waveform mean amplitudes and beta-values appeared consistent, with similar distributions across participants (Fig. 4b).

**Table 2:** Beta-values and waveform mean amplitudes for single condition activations in the auditory-ROI.

Condition	Beta-values (*p* value)	Waveform mean amplitude (*p* value)
Auditory speech-in-quiet	2.6 (< 0.001)	3.8 (< 0.001)
Auditory speech-in-noise	2.7 (< 0.001)	3.0 (< 0.001)
Tactile speech	1.2 (0.09)	1.7 (0.002)
Audio-tactile speech-in-noise	3.2 (< 0.001)	3.7 (< 0.001)
Control	− 0.05 (0.9)	− 0.4 (0.9)

Values are given in Δμmol/l with the respective FDR corrected *p* value in brackets.

### Contrasts

Audio-tactile gains were assessed by contrasting the *audio-tactile speech-in-noise* and *auditory speech-in-noise* conditions. *Audio-tactile speech-in-noise* elicited significantly larger HbO responses than *auditory speech-in-noise* in both the GLM analysis (beta difference = 0.6 Δμmol/l, *p* = 0.037) and the waveform averaging analysis (waveform mean amplitude difference = 0.7 Δμmol/l, *p* = 0.045), corresponding to an increase in HbO response of 19% and 23%, respectively. However, this effect did not exceed an additive criterion for multisensory integration. The summed responses of the *auditory speech-in-noise* and *tactile speech* conditions resulted on average in 0.28 Δμmol/l larger activations for waveform mean amplitudes (*p* = 0.01) and 0.7 Δμmol/l larger activations for beta-values (*p* = 0.06) compared to the *audio-tactile speech-in-noise* condition. These results indicate a sub-additive to additive enhancement effect at the group level. Individual audio-tactile gains from waveform averaging and GLM analyses are shown in Fig. 4c.

## Discussion

In the present study, we demonstrated that previously described audio-tactile advantages in speech understanding^19^ can be attributed to the influence of tactile stimuli on auditory neuronal processing. Using a dense optode montage, we first identified channels with the strongest responses to auditory sentences presented in quiet. The identified ROI comprised the middle and superior temporal gyri, in line with well-established literature on brain regions involved in speech processing^47,56,90,91^. We then found that vibrotactile speech cues enhanced cortical auditory responses in these channels, providing the first evidence of tactile enhancement effects on auditory speech processing using fNIRS. While *audio-tactile speech-in-noise* elicited larger responses than *auditory-only speech-in-noise*, the observed audio-tactile gains did not exceed the sum of unisensory responses, a criterion traditionally used to determine multisensory integration. Our result may reflect a near-saturation of auditory hemodynamic responses under unimodal stimulation, limiting the extent to which they can be further augmented by multimodal speech inputs.

### Individual differences in audio-tactile gains

The variability of individual audio-tactile gains as shown in Fig. 4c, underscores that despite the overall positive mean audio-tactile gains (sub-additive based on waveform averaging results, additive based on GLM results), not all participants exhibited stronger cortical oxygenation in the auditory ROI for audio-tactile compared to auditory-only speech.

Considering that all participants were normal-hearing, individual factors other than hearing status must have influenced the cortical processing of audio-tactile speech stimuli. Various factors such as tactile sensitivity, neural connectivity, prior experience with audio-tactile stimuli (e.g. from playing an instrument) or psychological aspects such as motivation or expectation may contribute to the magnitude of audio-tactile gains. It is likely that the amount of attention directed to the tactile stimulus significantly influenced the observed audio-tactile gains, which was not assessed in the present study. Thus, the extent to which participants focused on the concurrent vibrotactile input was uncontrolled and may have varied. Other studies have ensured attention to both stimulus modalities by asking participants to detect vibrotactile patterns and answer comprehension questions^18^, but have not tested whether performance in detecting tactile patterns correlates with auditory-tactile speech understanding or hemodynamic audio-tactile gains.

Moreover, it should be noted that waveform averaging and GLM analysis yielded highly comparable audio-tactile gains at the group level in this study (Fig. 4b). However, in only 10 out of the 21 participants did both methods show the same direction of effect. The fact that half of the participants did not show a consistent direction of effect across both methods suggests that the method chosen can significantly impact the results. Other studies confirm this replication issue for fNIRS data analyzed with different approaches^92^. Generally, the GLM approach is considered more robust to noise and false positives, as unexplained variance is accounted for by a noise term.

### Considerations of the experimental paradigm

This experiment was designed to identify cortical responses to auditory, vibrotactile and audio-tactile speech stimuli using fNIRS. To avoid distortion of the responses of interest, we aimed to minimize speaking artifacts, and therefore, did not record data on the participants’ speech understanding performance. Nevertheless, there is a tradeoff between active and passive task paradigms. Active tasks allow for monitoring of participants’ attention and tend to elicit stronger responses^93^, as has been shown for both, listening tasks and also somatosensory perception^94^. However, they often create artificial scenarios and require additional cognitive processes, such as memory consolidation, recall, and motor responses (e.g., button presses), which can introduce false positives due to event-locked motion artifacts. Passive listening paradigms more closely mirror natural listening conditions. They are preferred for objective assessments because they can be applied to a broader range of participants, including those who may not be able to provide active responses.

Here participants were instructed to listen to the speech carefully to understand what was being said, but we cannot rule out that the choice of a passive paradigm may have compromised participant engagement and overall effect sizes. Overall, the trade-off between active and passive task paradigms needs to be carefully evaluated with respect to the research questions being addressed and the intended use of the results, e.g. in future clinical applications targeting specific patient groups. Including an active paradigm and additional tactile conditions would allow assessment of whether our results mirror behavioral enhancements effect found for congruent but not incongruent^16,17^ or neutral^19^ tactile speech stimuli.

It should be noted that we tested for automatically evoked audio-tactile effects in cortical processing, as participants had no prior experience with tactile speech stimuli. The inclusion of a familiarization or training phase prior to the fNIRS recordings might have resulted in larger effects.

Unlike in our previous experiment where we used sound field presentation^19^ and could easily align the spatial direction of the auditory and tactile stimuli, spatial congruency was not explicitly addressed in the present study. Here, auditory stimuli were delivered through insert earphones, which did not create a binaural experience of the sound coming from the same direction as the tactile input, presented to the right index fingertip. Spatial congruency for multisensory integration has been attributed less importance than temporal congruency, but it still might have lowered the likelihood of integration of both signals^95,96^.

### Activation of auditory areas by tactile stimulation

The described audio-tactile gain suggests that the vibrotactile speech stimulus activated brain regions typically identified as auditory*.* That tactile stimuli activate auditory areas is conceivable via known cortico-cortical^97,98^ and thalamocortical^99^ connections, and has been repeatedly documented in the literature^100–102^. Multisensory processing in auditory areas was found particularly relevant for processing temporal stimulus features^103,104^. For example, Bolognini et al.^105^ demonstrated a causal role of the contralateral superior temporal gyrus for a tactile temporal discrimination task. With respect to vibrotactile speech stimuli, trained perception of sixteen-channel vocoded vibrotactile words presented on the forearm, produced similar fMRI responses in temporal lobes as auditory speech^38^. Remarkably, this result was present only for the vocoded vibrotactile stimulus that preserved temporal speech cues, while a token-based vibrotactile signal that led to similar behavioral performance in tactile word recognition did not show the same neural responses and changes in auditory-somatosensory connectivity. These results demonstrate a general ability of speech-like processing of a vibrotactile stimulus conveying temporal envelope information and suggest metamodal engagement of auditory areas^106,107^. Rather than responding selectively to auditory speech stimuli, temporal cortices seem to be sensitive to temporal structure and may be modulated also through heteromodal inputs^4^.

To ensure that noise from the shaker was not audible and did not evoke an auditory response, additional investigations were conducted (see Supplementary material). The results indicate that an actual auditory response in the tactile conditions is highly unlikely and cannot explain the observed audio-tactile gains in the auditory ROI.

While tactile processing in auditory regions remains well conceivable, the limited spatial accuracy of fNIRS complicates the separation of secondary somatosensory from auditory activation. Vibrotactile cues presented to the right fingertip are known to be processed in the left primary somatosensory cortex, from where input is projected further to bilateral secondary somatosensory cortices, located in the parietal operculum, which lies directly opposite to the primary auditory cortex in Heschl’s gyrus as part of the temporal operculum^108^. Neuroimaging studies have verified activations following this somatosensory pathway^109^. In accordance, our single channel results of the *tactile speech* condition show responses in left somatosensory and bilateral temporal areas (Fig. 2). Whereas primary somatosensory cortex activity can be mapped to activation in distinct channels in postcentral gyrus (e.g. S11-D9, S11-D12, S11-D11, S6-D3, see Fig. 3) responses in channels located over superior temporal gyri could be attributed to both auditory and secondary somatosensory areas.

Nonetheless, it is unlikely that responses in several channels over temporal cortices could be due solely to secondary somatosensory cortex activity. Even the most inferior channel S5-D2 located over right middle temporal gyrus showed activation in response to tactile speech alone (Fig. 2), pointing towards activation of speech-sensitive areas by the tactile stimulus rather than a secondary somatosensory cortex contribution, even when *tactile speech* was presented without auditory input.

**Fig. 5:**
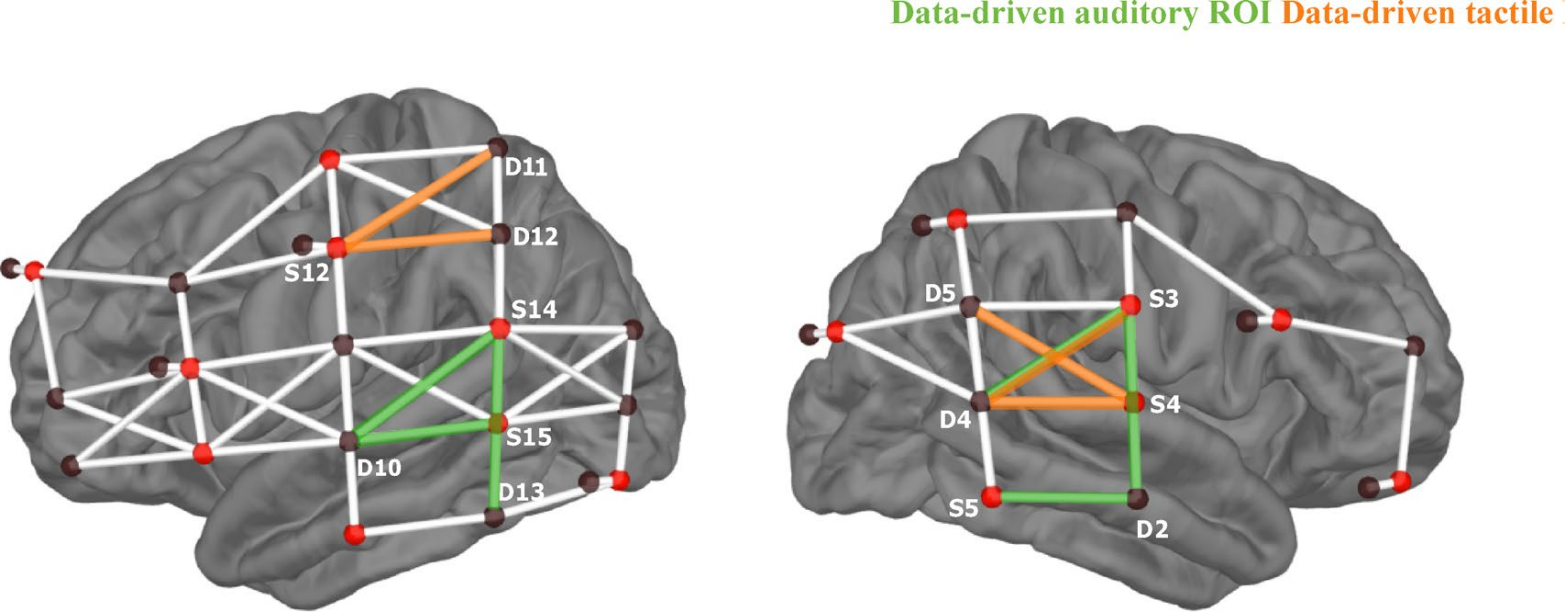
Auditory-ROI and tactile-ROI. Both ROIs were defined in a data-driven approach based on channels responding most consistent with the largest HbO amplitudes across the participant group. With the exception of one channel (S3-D4), both ROIs revealed distinct channel locations that can be attributed to somatosensory vs. auditory processing. S = Source, D = Detector.

To further verify whether the obtained audio-tactile gain could be interpreted as enhancement in auditory areas, we conducted the same procedure used to determine the *auditory-ROI*, using the *tactile speech* condition. This way, we obtained a frequency count for channels responding most often among participants to *tactile speech*, allowing us to explore whether different channels emerged with the highest preference for *tactile speech* compared to *auditory speech-in-quiet* stimuli. Contralateral (left) primary somatosensory cortex activation and bilateral activation in secondary somatosensory cortices are expected in response to right fingertip stimulation^110–112^. We identified a group of channels comprising five channels, distributed across left primary somatosensory cortex and right superior temporal gyrus, that responded most consistently to *tactile speech* in our participant group (S12-D11, S12-D12, S4-D5, S4-D4, S3-D4; see Fig. 5). This pattern may contrast with the results shown in Fig. 2, where bilateral activation of secondary somatosensory areas was observed in response to tactile speech. However, activation magnitude in the left-hemispheric channels was lower (Fig. 2), which likely explains why these channels did not emerge among the most responsive channels in this follow-up analysis (Fig. 5). The right-lateralized responses observed could be due to an optode placement that was more sensitive to the right secondary somatosensory cortex, despite similar levels of bilateral neural activation.

Alternatively, there might be a genuine right-hemispheric lateralization in the processing of vibrotactile speech stimuli. This question remains open for future research.

Importantly, within the identified *tactile ROI*, only channel S3-D4 overlapped with the earlier defined *auditory-ROI*, indicating that auditory-sensitive areas are not the same as tactile-sensitive areas. This follow-up analysis thus supports the interpretation that the observed audio-tactile gain reflects a genuine enhancement effect in auditory regions with minimal to no contribution from purely somatosensory processing areas.

Modulations by projections from other areas or the inherent multimodality of the auditory-ROI may underlie this effect. The latter is often referred to as “piggybacking”, where somatosensory input is “piggybacked” onto auditory processing, assuming a fundamentally modality-neutral nature of speech areas^113^. This hypothesis was supported by recent observations demonstrating a dominance of audio-tactile multisensory neurons across all cortical layers, justifying the designation of temporal cortices as inherently multisensory^114^. However, similar cross-modal activations have been found to be driven by altered sensory excitability in deep anesthetic states^115^, making this finding questionable. Possible bottom-up versus top-down modulations contributing to the effects will be discussed in the next section.

### Vibrotactile influences on auditory cortical activations are likely mediated by top-down modulations

The presented findings speak in favor of an actual influence of the vibrotactile stimulus on processing in auditory regions. However, audio-tactile gains did not exceed an additive criterion. Often, true multisensory integration is interpreted as bottom-up sensory interaction, inferred if the response magnitude in the multisensory condition significantly deviates from the unisensory activation, e.g. significantly exceeds or suppresses the sum of unisensory responses^116^. While originally defined for spike counts of single neurons in feline superior colliculus, multisensory integration principles have been adopted also to fMRI and fNIRS measurements^61,117–119^. We anticipated audio-tactile gains (instead of suppressions), because previous fMRI and fNIRS investigations found enhancements for audio-visual speech and audio-tactile non-speech stimuli^121^. Similar to our findings, other fNIRS investigations that assessed MSI effects neither observed multisensory integration effects based on superadditivity nor the principle of inverse effectiveness^56,60,61^.

Inferring multisensory integration as defined for firing rates of single neurons from fNIRS measurements is generally problematic because they constitute only indirect measures of a large number of neural populations. Instead of spike counts on an ordinal scale, relative oxygenation changes influenced by the metabolism of millions of neurons are measured on an interval scale with a normalized baseline. Consequently, linear summation may not be directly applicable. Even when cortical activations exceed a certain multisensory integration criterion (whether maximum or additive), the measured response could arise from metabolic changes driven by multisensory neuronal populations as well as co-activated unisensory neurons^118,122^. Hence, multisensory thresholds may be considered as a loose indicator for multisensory integration rather than its proof. Its absence as observed in our case, can either be attributed to a saturation effect or argues against true bottom-up integration effects. The latter case would suggest a stronger contribution of top-down influences in driving tactile activations in auditory areas. These are likely modulations from somatosensory regions via cortico-cortical connections^98^ or top-down attentional influences^123^. It is known that feedback projections from higher order associative areas dominate outer cortical layers^124–126^ and that the concentration of these feedback connections is reflected in cortical thickness^127^. fNIRS, measuring from superficial cortical areas may thus record activation related to feedback projections with higher sensitivity than those elicited by feedforward projections. Hence, it might also be that afferent auditory and tactile information is integrated in deeper cortical layers and was not detected.

A bottom-up processing of vibrotactile speech in auditory areas may not be far-fetched, as the same tactile stimulus could be perceived by the auditory system if it was transmitted through air and picked up by the ear instead of presented to the skin. Early metamodal processing might be more natural for auditory and tactile speech stimuli than for e.g. visual facial speech cues which are transmitted through light and constitute a different form of energy at a completely different timescale. Thus, processing of tactile stimuli in an auditory fashion is conceivable from the lowest levels, while visual speech may only mimic auditory speech processing at higher hierarchical levels, such as when reaching phoneme (and viseme) representations.

For non-speech audio-tactile stimuli, bottom-up sensory integration has been localized in superior temporal gyrus, contralateral to the side of tactile stimulation presentation^128^. We did not contrast left and right-hemispheric effects in our study, but measured multisensory gains in a bilateral auditory ROI, which may have blurred a potentially stronger right-hemispheric integration effect.

Considering single channel activations elicited by separate conditions (Fig. 2), a more pronounced right-hemispheric temporal activation elicited by *tactile speech* and a left-hemispheric dominance in *auditory-only* conditions seemed to disappear for *audio-tactile speech*. While often the left hemisphere appears to be dominant in speech processing^129,130^, particularly slow features of speech, such as syllabic processing have been attributed to right auditory areas^131,132^. Note that lateralization effects may differ between individuals (e.g. dependent on sex^133,134^, which was neglected in our analysis. It was surprising that only right and not bilateral STG channels were identified in the data-driven tactile ROI as expected for vibrotactile stimulation^94,135^. This may be due to a right-hemispheric dominance of SII processes^136^ or a montage placement that measured with higher specificity from right than left SII. Alternatively, the responses to tactile speech may reflect not purely secondary somatosensory cortex activation, but partly right-hemispheric speech envelope processing in auditory areas triggered by the tactile stimulus alone. A right-hemispheric lateralization has also been described for theta oscillatory activity associated with the integration of syllabic units^137^.

That tactile envelope cues aid in parsing a continuous speech signal into words and syllables has been hypothesized as a mechanism behind benefits in speech understanding as reported in our and other previous studies^12,14,17–19^. Top-down connections from secondary somatosensory or higher order associative areas may mediate the enhancements in speech understanding, particularly in normal-hearing participants (review in Kral and Sharma^4^). The temporally aligned vibrotactile input may entrain attention in a temporally tuned manner towards the incoming speech stimulus, thereby facilitating linguistic parsing. Various literature indicates that such a process could be influenced by top-down projections^138–140^ from, among others, prefrontal areas^141–144^ and be accompanied by a synchronization of low-frequency cortical oscillations as has been reported for top-down attentional mechanisms^141,145^, in auditory processing^146^ and audio-tactile integration^18,36^.

Next to top-down influence by temporal modulations, prior knowledge of the fact that vibrotactile cues provide speech information may have consciously or unconsciously led to an interpretation of the tactile stimulation as being auditory. Exposure to preceding auditory speech stimuli, paired with the congruent *tactile speech* stimulus may have caused a priming effect, so that participants anticipated or imagined a corresponding auditory signal. The imagination of a sound percept can elicit comparable activations to real stimulation, as shown for musical auditory imagination^147,148^. In that scenario, vibrotactile speech would not be processed in auditory areas by a bottom-up process but would indirectly trigger (for tactile input only) or reinforce (for audio-tactile input) an auditory percept via mental imagery that was reflected in enhanced cortical oxygenation changes in auditory areas by top-down connections from frontal cortices. Indeed, some participants reported after the experiment that they imagined what kind of sentences the signals could have been related to, supporting an auditory interpretation of tactile cues by top-down and context-dependent influence which likely contributed to the observed audio-tactile gains.

Multisensory gains are generally assumed to be driven by a combination of top-down and bottom-up processing. We assume that also here, both early vibrotactile interactions in auditory areas, along with attentional modulations that inform about syllable onsets in a top-down manner, are mechanisms that contributed to the obtained effect. Possibly, the audio-tactile gain was further reinforced by conscious or unconscious interpretations of the vibrotactile stimulus as auditory speech.

### Conclusion

The present study showed that a subtle, congruent vibrotactile signal presented to one fingertip elicited measurable cortical gains in auditory areas compared to the auditory signal alone. The observed effects may be explained by attentional modulations enhancing perceptual focus on temporally relevant speech segments, modulations from somatosensory cortices, and learned associations of tactile cues with auditory speech sounds.

### Outlook

Understanding the neural basis of audio-tactile speech enhancements will help to design more effective tactile stimuli and advance the development of tactile aids. These could be particularly supportive for hard of hearing or deaf individuals who are unable to access cochlear implants or experience unsatisfactory outcomes of their hearing treatment. Further applications are conceivable during language acquisition^149^ and cochlear implant rehabilitation, second language learning, or for individuals with dyslexia^150^.

## Electronic supplementary material

Below is the link to the electronic supplementary material.

Supplementary Material 1

## Data Availability

The dataset generated in this study is not publicly available due to data protection issues but can be made available from the corresponding author on reasonable request.
