# Supplementary material for: Vibrotactile speech cues are associated with enhanced auditory processing in middle and superior temporal gyri
Source: Sci Rep. 2025 Jul 12;15:25202. doi: 10.1038/s41598-025-07718-8 (PMC12255685; doi:10.1038/s41598-025-07718-8)
Supplement: Supplementary file 1 — Supplementary Material 1 [file 41598_2025_7718_MOESM1_ESM.docx]

# Supplementary materials

## Preprocessing


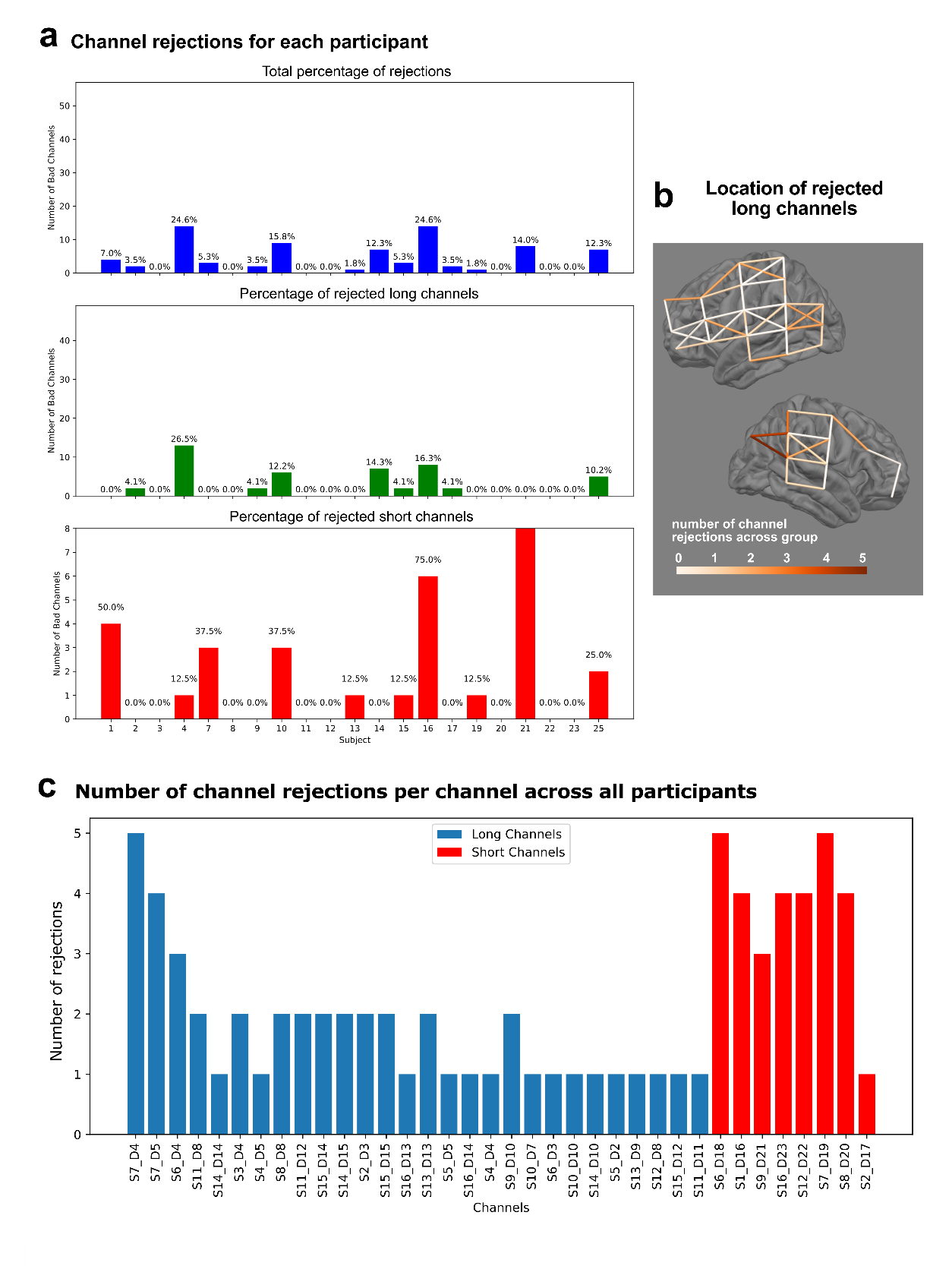


**Figure S1. Number of rejected channels**. **a**. For each participant, the total number of channel rejections (out of 56) (top), the number of rejected long channels (out of 46) (middle), and the number of rejected short channels (out of 8)(bottom) are displayed. **b.** Distribution of rejected long channels on the montage. The number of rejected long channels refers to the number of participants (out of 21) for which that specific long channel was excluded. **c.** Bar plot showing the number of rejections across the group, including also short channels (shown in red).


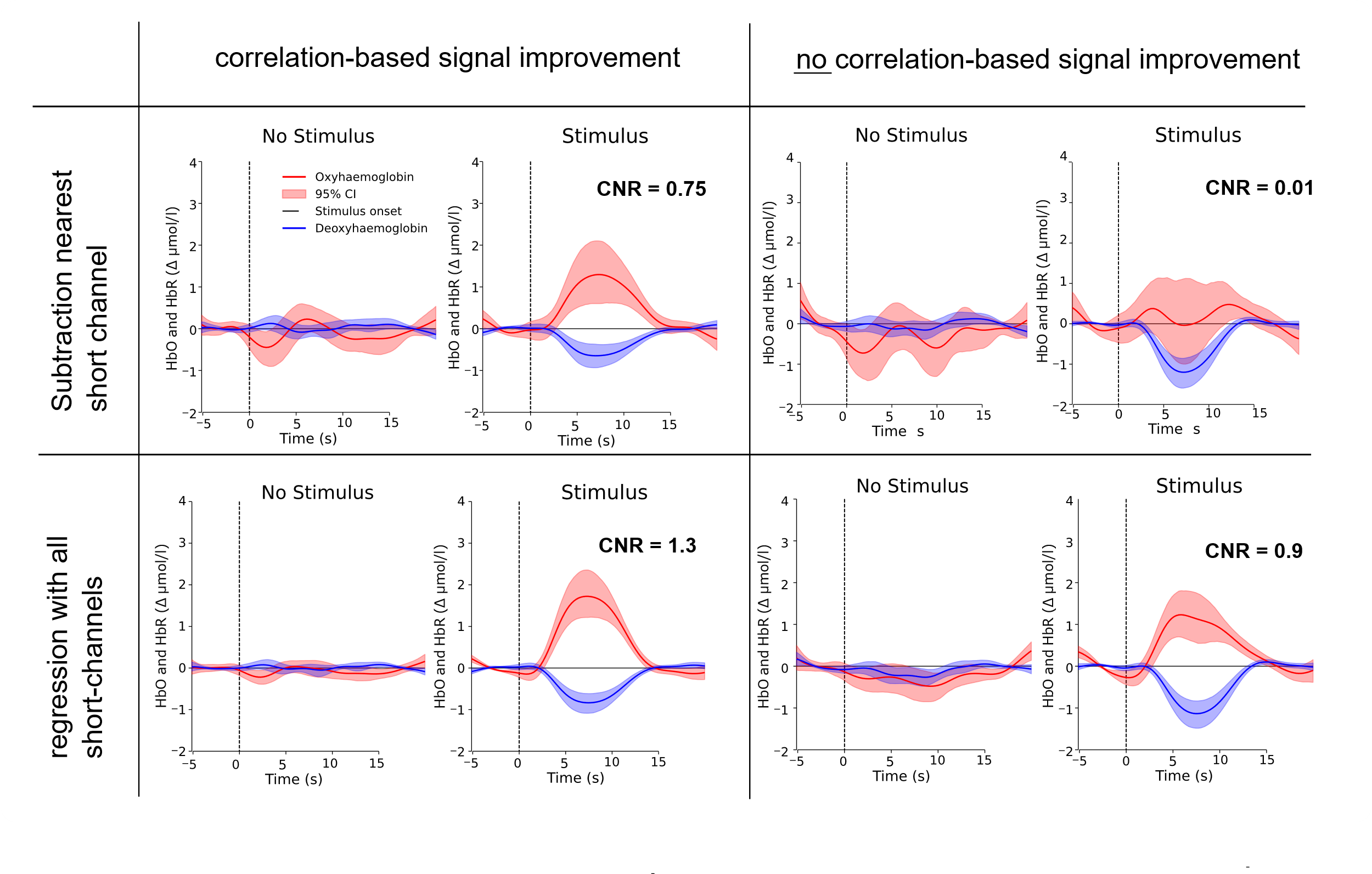


**Figure S2. Effects of short-channel correction method and correlation-based signal improvement.** Improvements in contrast-to-noise ratios (CNRs) of waveform responses for the ‘Stimulus’-condition were found when employing short-channel regression in a separate regression step with all short-channel traces included as regressors, compared to a subtraction method based on the nearest short-channel only. Moreover, CNRs for the ‘Stimulus’-condition improved when applying CBSI. The ‘Stimulus’-condition comprises all seven task conditions grouped together, while ‘No Stimulus’ included the two control conditions for which no evoked response is expected. We saw significant improvements in CNR in the stimulus conditions but no changes in the control condition.The contrast-to-noise ratio (CNR) was calculated similar as in Zhou et al. (2020): CNR = mean(HbO_sig_)/sqrt(std(HbO_sig_)^2^ + std(HbO_noise_)^2^ , with HbO_sig_ referring to the mean HbO of samples around the expected peak-time, between 5 and 8s. HbO_noise_ refers to the 5 s of baseline. This analysis informed our choice of applying short-channel correction with all short-channels in a separate regression step and including CBSI, as this combination yielded the largest CNR of 1.3 in the ‘Stimulus’- condition without changing the null results in the ‘No stimulus’- condition (lower left).

## Potential impact of auditory stimulation from shaker noise on auditory cortex responses in the tactile conditions appears to be negligible

Another explanation for the activation of auditory areas in response to tactile stimulation could be actual auditory stimulation caused by the low-frequency sound elicited by vibrations of the shaker, despite participants wearing insert earphones with 30+dB external noise exclusion (https://www.interacoustics.com/images/research/pdf/ER-2%20data%20sheet.pdf) and the surrounding masking noises of the fNIRS system and room ventilation being of similar levels. The contribution of the shaker caused an increase of 3 dB SPL LAF on the average spectrum (from 33 to 35 dB SPL LAF), with a 7dB SPL LZeq difference at 250 Hz (which was the closest to the 230 Hz signal of the available analyzed frequencies). It is unlikely to see reliable fNIRS responses for tones close to threshold levels^1,2^. Nonetheless, we wanted to explore the potential audibility of the shaker further.

Based on participants’ reports and our own experience, it is difficult to determine whether one actually heard or automatically imagined sound accompanying the vibrotactile signal. In a few participants, we explored the audibility of the shaker noise without touching it by using a two-alternative-forced-choice task following the main experiment. In this task, participants indicated whether a stimulus was presented on the shaker either in the first or second time window. Four out of nine participants tested did not perceive any sound from the shaker (responses not significantly different from chance), five out of nine participants were able to indicate correctly above chance level when the shaker was producing a *tactile speech* stimulus. These mixed results did not show a correlation with audiogram thresholds and are most likely attributable to differences in the earplug fit within the ear canal or perhaps higher level auditory or cognitive processing abilities. While these findings suggest that the shaker noise was near the threshold of detectability, this follow-up experiment differed in important ways from the main experiment: it was a unimodal detection task in which participants were asked to listen explicitly for shaker noise, without touching the shaker. Consequently, it cannot fully answer the question of whether an auditory percept driven by bottom-up auditory sensory stimulation may have occurred during a multimodal condition as tested in the main experiment. In the setup of the main experiment interactions between auditory and tactile stimuli could have influenced auditory thresholds and loudness percepts^3^.

To address this issue, we included a second tactile speech condition with continuous background noise in the experiment for a subset of six participants. This allowed us to compare cortical responses to tactile speech in silence (*tactile speech*) versus *tactile speech-in-auditory-noise*. No significant differences were found across four predefined ROIs covering auditory and somatosensory regions. These effects were absent in both waveform mean amplitudes nor in beta-values (see **Figure S2** and **Table 3**). The findings rule out any measurable differences in relation to audibility to the shaker (at least in a group of six participants).

Although we cannot prove the absence of an acoustic percept from the shaker noise, several steps were taken indicating that activation in temporal areas due to auditory input in the tactile condition is unlikely or negligibly small.

**Table 3. Contrast of tactile speech in silent background versus tactile speech in masking noise.** Cortical responses to tactile speech stimuli without background noise as used in the reported experiment were compared to an additional tactile speech in masking noise in four predefined regions-of-interest (ROI) targeting left somatosensory cortex (left SC), right somatosensory cortex, left temporal cortex, and right temporal cortex. A linear mixed effects model with the interaction between ROI and condition as main effect and participants as random effects was conducted once for beta-values of the ROI GLM results, and once for mean waveform amplitudes. Values are given in Δμmol/l with the respective FDR corrected p-value in brackets.

| **ROI** | **Beta-values (p-value)** | **Waveform mean amplitudes (p-value)** |
| --- | --- | --- |
| Left somatosensory cortex | -0.117 (0.8) | 1.042 (0.5) |
| Right somatosensory cortex | -0.121 (0.8) | 1.385 (0.4) |
| Left temporal cortex | 0.388 (0.8) | 0.472 (0.7) |
| Right temporal cortex | 0.128 (0.8) | 0.781 (0.6) |


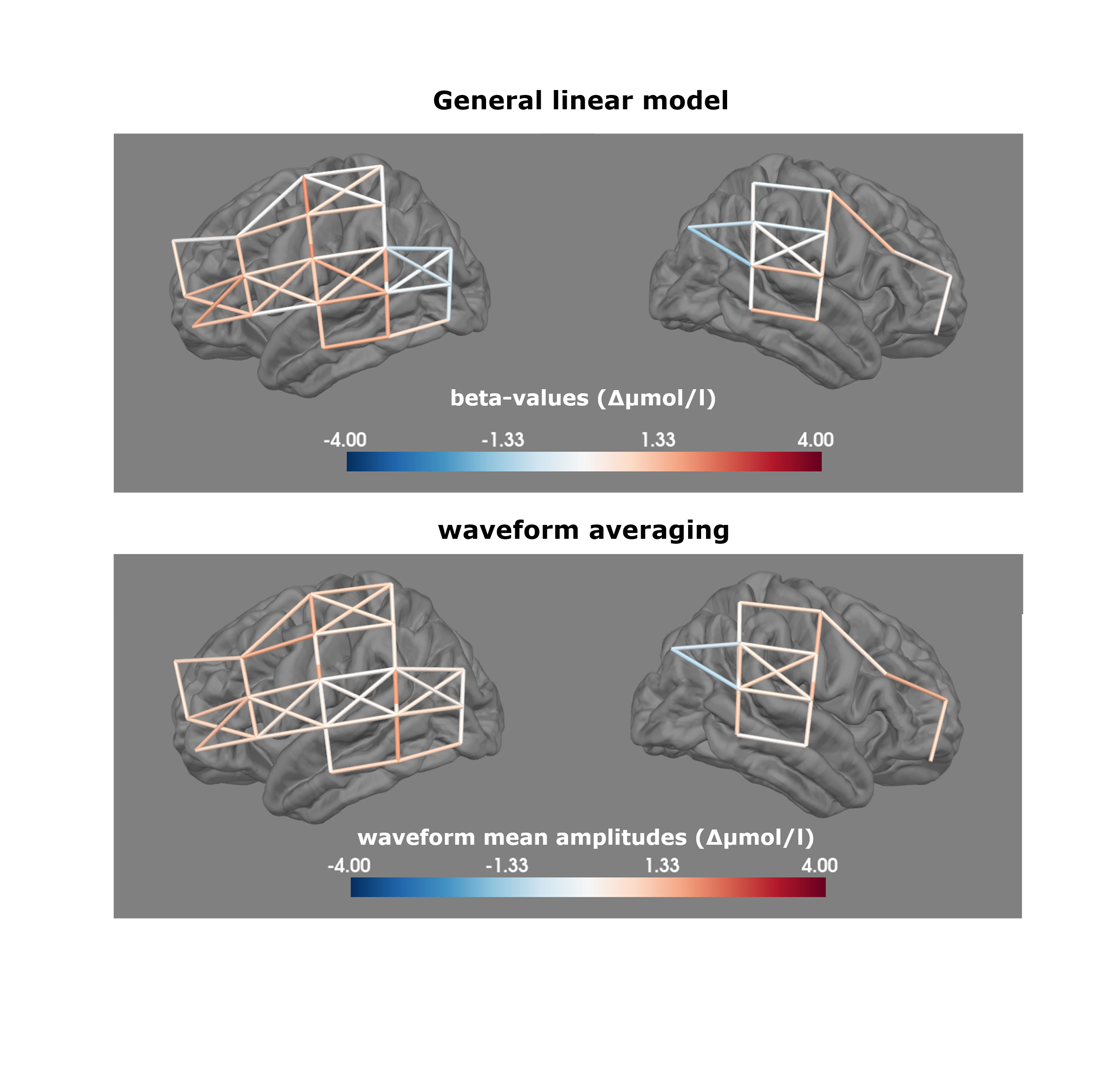


**Figure S3. Contrasts tactile speech in silence vs. tactile speech in noise.** A model of the form HbO response ~ Condition:Channel + 1|Participant with ‘tactile speech in silence’ and ‘tactile speech in noise’ as conditions was conducted for beta-values (top) and waveform mean amplitudes (bottom). No channel reached significance (p<0.05).

# References supplementary information

1. Weder, S. *et al.* Cortical fNIRS Responses Can Be Better Explained by Loudness Percept than Sound Intensity. *Ear Hear* 41, 1187–1195 (2020).

2. Weder, S., Zhou, X., Shoushtarian, M., Innes-Brown, H. & McKay, C. Cortical Processing Related to Intensity of a Modulated Noise Stimulus—a Functional Near-Infrared Study. *JARO - Journal of the Association for Research in Otolaryngology* 19, 273–286 (2018).

3. Yarrow, K., Haggard, P. & Rothwell, J. C. Vibrotactile – auditory interactions are post-perceptual. *Perception* 37, 1114–1130 (2008).
